# Associations of healthy eating index 2020 and its components with non-alcoholic fatty liver disease in type 2 diabetes patients and the mediating roles of metabolic indicators: NHANES 2007–2018

**DOI:** 10.3389/fnut.2025.1564197

**Published:** 2025-04-09

**Authors:** Jingxiong Chen, Haizhou Diao, Yuling Zhang, Ben Hu, Kai Qian, Kaiguang Zhang, Tengyue Zhang, Jizhong Song

**Affiliations:** ^1^Department of Postgraduates, Bengbu Medical University, Bengbu, China; ^2^Department of Gastroenterology, The First Affiliated Hospital of USTC, Division of Life Sciences and Medicine, University of Science and Technology of China, Hefei, China; ^3^Department of Oncology, The First Affiliated Hospital of USTC, Division of Life Sciences and Medicine, University of Science and Technology of China, Hefei, China; ^4^Department of Gastrointestinal Surgery, The First Affiliated Hospital of USTC, Division of Life Sciences and Medicine, University of Science and Technology of China, Hefei, China; ^5^Department of Cardiology, The Second People’s Hospital of Hefei, Hefei Hospital Affiliated to Anhui Medical University, Hefei, China

**Keywords:** non-alcoholic fatty liver disease, type 2 diabetes mellitus, healthy eating index 2020, metabolic indicators, NHANES, dietary quality

## Abstract

**Background:**

Non-alcoholic fatty liver disease (NAFLD) represents a major public health issue, especially among individuals diagnosed with Type 2 diabetes mellitus (T2DM), where its prevalence can reach up to 70%. This research examines the relationship between the Healthy Eating Index 2020 (HEI-2020) and its individual components with the occurrence of NAFLD in T2DM patients, while also investigating the potential mediating effects of various metabolic indicators.

**Methods:**

Data from the National Health and Nutrition Examination Survey (NHANES) spanning 2007 to 2018 were utilized. This cross-section study included 1,770 T2DM patients, who were divided into NAFLD and non-NAFLD groups using the Fatty Liver Index as a diagnostic tool. The HEI-2020, which assesses diet quality, was computed based on 24-h dietary recall data. Key metabolic indicators such as the triglyceride-glucose (TyG) index, metabolic score (MS), mean arterial pressure, uric acid levels, and total cholesterol were evaluated.

**Results:**

The findings indicated that higher HEI-2020 scores were associated with a lower likelihood of NAFLD (odds ratio 0.978, 95% confidence interval: 0.959–0.998), with the strongest inverse associations observed in the top quartiles of diet quality. Whole fruits, greens and beans, and saturated fat were crucial dietary factors. Mediation analysis demonstrated that the TyG index and MS accounted for 5.11 and 36.94% of the relationship between HEI-2020 and NAFLD, respectively.

**Conclusion:**

Greater adherence to the HEI-2020 is associated with a lower likelihood of NAFLD in T2DM patients, with metabolic indicators partially mediating this association. Enhancing diet quality, particularly by increasing the consumption of whole fruits and greens while reducing saturated fat intake, may be important in managing metabolic health and liver function in this vulnerable population.

## 1 Introduction

Non-alcoholic fatty liver disease (NAFLD) has become a significant global health issue, particularly among individuals diagnosed with Type 2 diabetes mellitus (T2DM) (1). NAFLD is defined by the excessive accumulation of fat in the liver, unrelated to high alcohol consumption, and is strongly associated with insulin resistance, obesity, and metabolic syndrome (2). This condition often precedes more serious liver diseases, such as non-alcoholic steatohepatitis (NASH), fibrosis, and cirrhosis, thereby increasing the risk of liver-related morbidity and mortality (3). The prevalence of NAFLD in patients with T2DM is notably high, with studies indicating that up to 70% of these patients may be affected. Consequently, early intervention and management of NAFLD in T2DM patients are crucial (4, 5). However, most existing studies have primarily focused on the impact of traditional environmental factors (e.g., chemical exposures), and further research is needed to identify other contributing factors (6, 7). Given the increasing prevalence of both T2DM and NAFLD, identifying modifiable risk factors that could help slow the progression of these diseases is crucial (8).

Diet, a fundamental component of human life, is not only essential for survival but also plays a vital role in maintaining health (9). The ancient Chinese medical text, *Huangdi Neijing* (The Yellow Emperor’s Classic of Medicine), emphasizes the importance of a balanced diet, stating that “Grains are for nourishment, fruits are for assistance, livestock is for benefit, and vegetables are for supplementation.” Numerous epidemiological and clinical studies have shown that diet has a broad impact on health and disease, influencing conditions such as cardiovascular disease, diabetes, metabolic syndrome, neurological disorders, and cancer (9–12). In managing and preventing metabolic diseases, including T2DM and NAFLD, diet plays an essential role. Among various tools used to assess diet quality, the Healthy Eating Index (HEI) is a widely used measure. The HEI evaluates adherence to the Dietary Guidelines for Americans (DGA), and the most recent version, HEI-2020, considers multiple dietary components such as fruits, vegetables, whole grains, and refined grains (13). Higher HEI scores have been associated with improved overall diet quality and a lower risk of chronic diseases, including chronic respiratory disease and certain cancers (14–16). Recent studies suggest that better adherence to the HEI-2020 is associated with improved metabolic health, which may, in turn, be related to the development and progression of NAFLD, particularly in individuals with T2DM.

Despite growing interest in the role of diet in metabolic disorders, few studies have specifically examined the relationship between the HEI-2020 and NAFLD in T2DM patients. Additionally, the potential mediating role of metabolic indicators remains understudied. It has now been established that metabolic indicators are strongly associated with the occurrence, progression, and prognosis of various diseases (17–19). Total cholesterol (TC) and uric acid (UA) are commonly used as biomarkers for hyperlipidemia and hyperuricemia, respectively (20). Mean arterial pressure (MAP), which integrates systolic and diastolic blood pressure, serves as an indicator of hypertension and addresses the challenges of using both measurements in statistical models (21). Metabolic scores (MS) help identify individuals with abnormal metabolic levels (22). The triglyceride-glucose (TyG) index, a marker of metabolic dysfunction, has emerged as a reliable indicator of insulin resistance and a predictor of NAFLD (23, 24). These metabolic indicators play a crucial role in the pathophysiology of both T2DM and NAFLD. Understanding their interactions could shed light on how diet is associated with liver health in this population. Therefore, investigating whether the HEI-2020 can mediate or modify the relationship between metabolic dysfunction and NAFLD in T2DM patients by improving diet quality is of significant importance.

In this research, we intend to leverage data from the National Health and Nutrition Examination Survey (NHANES) spanning the years 2007 to 2018 to examine the relationships between the HEI-2020 and its individual components with NAFLD in patients diagnosed with T2DM. Furthermore, we will investigate the potential mediating effects of key metabolic indicators, such as the TyG index (23), MS (22, 25), MAP (20), UA, and TC, in the relationship between diet quality and NAFLD. This study is pivotal for deepening our comprehension of how dietary habits impact liver health in individuals with T2DM and for uncovering potential dietary strategies to alleviate the prevalence and severity of NAFLD within this vulnerable population.

## 2 Methods

### 2.1 Study design and population

This study utilized data derived from the National Health and Nutrition Examination Survey (NHANES), a cross-sectional survey that is nationally representative and administered by the Centers for Disease Control and Prevention (CDC) in the United States (26). HANES is designed to collect extensive health and nutritional information through a combination of laboratory analyses, physical examinations, and structured interviews, providing a comprehensive overview of the health conditions prevalent among the U.S. population. The survey employs a sophisticated, multistage probability sampling methodology to ensure that the collected data accurately reflect the demographics of the civilian, non-institutionalized population.

**Figure 1:**
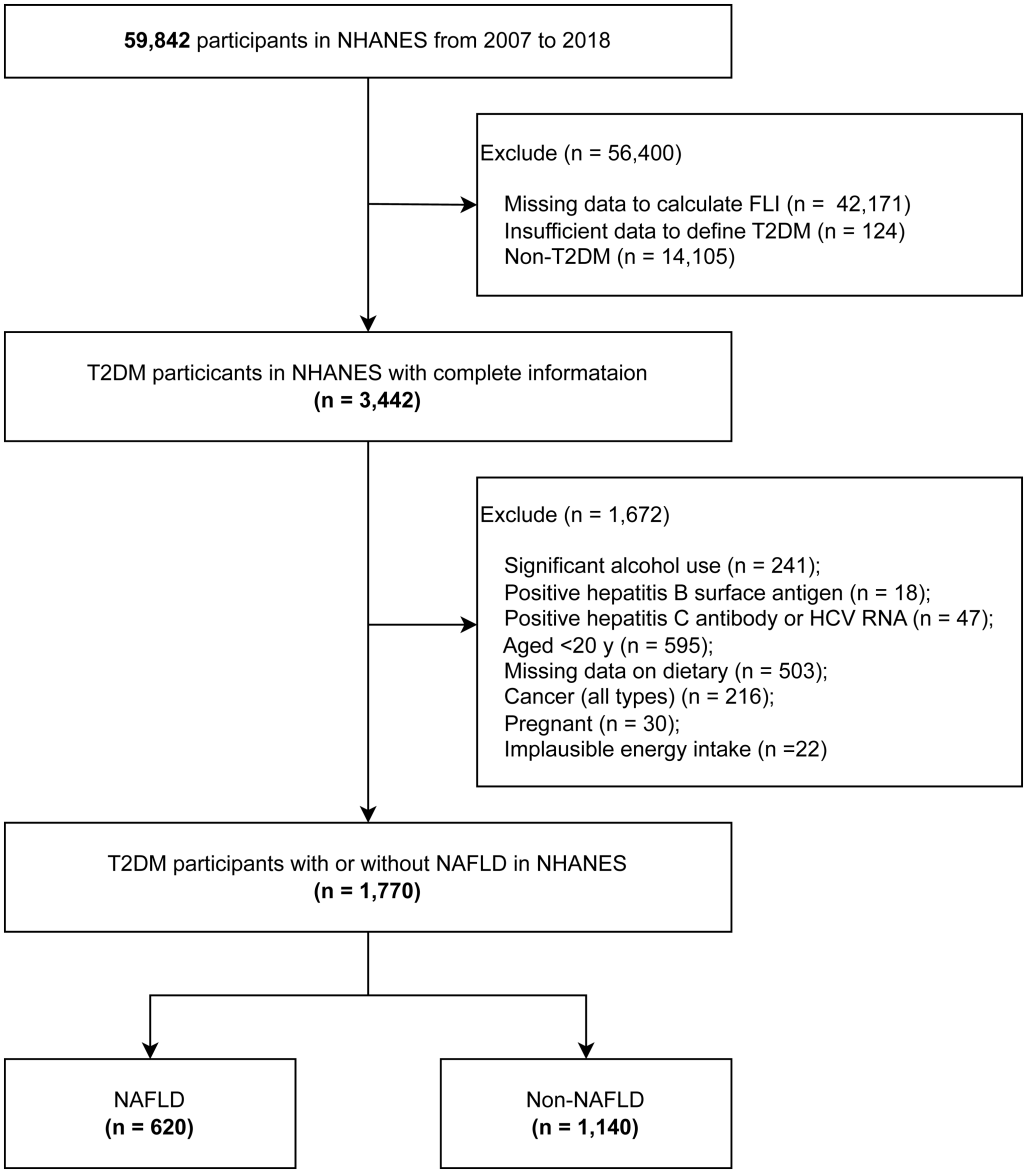
The flowchart of analytic sample selection.

For the purpose of this analysis, data from six sequential NHANES cycles (spanning from 2007 to 2008 to 2017–2018) were consolidated to enhance the sample size and strengthen the statistical robustness of the research. Among the 59,842 NHANES participants from 2007 to 2018, the Fatty Liver Index (FLI) was calculated for each individual. T2DM was defined as meeting any one of the following criteria: (1) blood glucose levels surpassing 11.1 mmol/L during an oral glucose tolerance test; (2) fasting blood glucose levels exceeding 7.0 mmol/L in laboratory tests; (3) HbA1c levels greater than 6.5%; (4) random blood glucose levels exceeding 11.1 mmol/L; (5) use of diabetes medications; or (6) self-reported diagnosis of diabetes. Individuals with Type 1 diabetes, defined as those diagnosed before the age of 30 and exclusively using insulin for glycemic control, were excluded. After excluding participants with significant alcohol consumption or those who tested positive for hepatitis C antibody, hepatitis C virus RNA or hepatitis B surface antigen, the remaining participants were categorized into two groups: NAFLD and non-NAFLD. Further exclusions were made for participants younger than 20 years, those with any type of cancer, those with unavailable or unreliable dietary information, pregnant individuals, and those with implausible energy intake (men: >8,000 kcal/day, women: >5,000 kcal/day, or < 500 kcal/day) (27, 28) were further excluded. Ultimately, the analysis included 1,140 participants with NAFLD and 630 participants without NAFLD within the T2DM cohort. A comprehensive flowchart detailing the selection process of the study population is presented in Figure 1. The research protocol received approval from the National Center for Health Statistics Research Ethics Review Board, and written informed consent was obtained from all participants involved in the study. The investigation adhered to the Strengthening the Reporting of Observational Studies in Epidemiology (STROBE) reporting guideline (29).

### 2.2 Measurement of healthy eating index 2020

The dietary assessment, referred to as *What We Eat in America*, was carried out utilizing the Automated Multiple-Pass Method (AMPM), a standardized and rigorously developed approach created through a collaborative effort between the U.S. Department of Agriculture (USDA) and the U.S. Department of Health and Human Services. The AMPM was specifically engineered to offer a systematic and dependable methodology for gathering dietary intake data in large-scale national surveys. Its accuracy and reliability have been substantiated through multiple validation studies (30–32).

Participants enrolled in the NHANES were required to complete two separate 24-h dietary recall interviews. These interviews were designed to meticulously document the types and quantities of foods consumed during the 24-h period preceding the interview, spanning from midnight to midnight. The initial interview was conducted face-to-face at the Mobile Examination Center (MEC), while the follow-up interview was administered over the phone, occurring between 3 and 10 days after the first session. The primary objective of these interviews was to gather exhaustive data on the diversity and quantities of foods consumed. The USDA’s Food and Nutrient Database for Dietary Studies (FNDDS) was employed to derive nutrient and food component values for all reported food items (33).

The HEI-2020 is a tool designed to assess diet quality based on adherence to the DGA 2020–2025. The HEI-2020 consists of 13 components, including nine adequacy components and four moderation components, which collectively reflect the key recommendations of the DGA (13). The adequacy components promote the consumption of beneficial food groups, such as protein foods, whole grains, dairy, vegetables and fruits. In contrast, the moderation components aim to restrict the consumption of less healthy dietary elements, including sodium, saturated fats, added sugars and refined grains. Each component is assigned a specific weighting and a maximum score, with the total HEI-2020 score ranging from 0 to 100. In this study, the HEI-2020 scores were computed using the two 24-h dietary recalls provided by participants. A higher score signifies superior diet quality.

### 2.3 Definition of NAFLD

Fatty liver was assessed using the FLI, calculated through the following mathematical formula:

$$\mathrm{where}FLI=e^{y}/\left(1+e^{y}\right)\times 100$$

y = (−0.8073 × non-Hispanic Black +0.3458 × Mexican American) + (0.0093 × age) + (0.6151 × loge *γ*-glutamyltransferase) + (0.0249 × waist circumference) + (1.1792 × loge insulin) + (0.8242 × logeglucose) − 14.7812.

In this equation, the variables were defined as follows: (1) Ethnicity: Non-Hispanic Black and Mexican American were assigned a value of 1 if the participant identified with that ethnicity, and 0 otherwise; (2) Age: Measured in years; (3) γ-glutamyltransferase: Measured in international units per liter (IU/L); (4) Waist circumference: Measured in centimeters (cm); (5) Insulin: Measured in picomoles per liter (pmol/L); (6) Glucose: Measured in milligrams per deciliter (mg/dL). The FLI threshold of 30 or higher was chosen to signify the presence of fatty liver due to its diagnostic accuracy. It exhibits an area under the receiver operating characteristic (ROC) curve of 0.80 (95% confidence interval [CI]: 0.77–0.83) for predicting ultrasound—confirmed NAFLD (34).

Moreover, we systematically excluded other identifiable causes of chronic liver disease. These exclusions included viral hepatitis, which was confirmed by positive diagnostic markers such as hepatitis C antibody, hepatitis C virus RNA, or hepatitis B surface antigen. Additionally, significant alcohol intake was ruled out. This was defined as women consuming more than one standard drink per day and men consuming more than two standard drinks per day. A standard drink was quantified as containing 14 grams of pure alcohol. These exclusions encompassed viral hepatitis, confirmed through positive diagnostic markers such as hepatitis C antibody, hepatitis C virus RNA or hepatitis B surface antigen. Additionally, significant alcohol intake was excluded, defined as consuming more than more than one per day for women or two alcoholic beverages per day for men (35). A standard drink was quantified as containing 14 grams of pure alcohol (36).

### 2.4 Metabolic indicators

Blood pressure assessments were conducted at the MEC. Fasting plasma glucose, triglyceride levels, TC, and UA concentrations were determined enzymatically using an automated analyzer. MAP was calculated by adding one-third of the difference between systolic and diastolic blood pressures to the diastolic pressure. TyG index was derived by computing the natural logarithm of the product of fasting triglyceride levels and fasting glucose levels, divided by two. Furthermore, the MS was developed by summing the z-transformed values of four key components: TC, UA, MAP, and the TyG index (21).

$$MS=Z\left(TC\right)+Z\left(UA\right)+Z\left(\mathrm{MAP}\right)+Z\left(TyG\right)$$

### 2.5 Covariates

Based on existing literature, factors that have been demonstrated to correlate with NAFLD and dietary quality were incorporated into the analysis. These factors encompassed demographic characteristics such as age, Body Mass Index (BMI), levels of physical activity, ethnicity, household income (measured using the poverty-income ratio, or PIR), educational background, and smoking status. Participants were categorized into three distinct income groups based on their income-to-poverty ratio: those with a ratio of less than 1.0, those with a ratio between 1.0 and 3.0, and those with a ratio exceeding 3.0. Smoking status was further classified into three categories: never smokers (defined as individuals who had smoked fewer than 100 cigarettes in their lifetime), current smokers (defined as those who had smoked at least 100 cigarettes in their lifetime and continued to smoke), and former smokers (defined as individuals who had smoked at least 100 cigarettes but had since quit). Physical activity levels were also assessed, with a high level of physical activity defined as exceeding 600 MET-minutes per week, while light physical activity was defined as 600 MET-minutes per week or less (37). BMI was segmented into three categories: less than 25.0, 25.0 to 29.9, and 30.0 or higher. Dyslipidemia was identified by the presence of at least one of the following criteria: a total cholesterol level of 200 mg/dL or greater, a low-density lipoprotein (LDL) cholesterol level of 130 mg/dL or higher, a triglyceride level of 150 mg/dL or above, or a high-density lipoprotein (HDL) cholesterol level below 40 mg/dL (38). Data on the consumption of dietary supplements over the previous 30 days was meticulously collected by trained professionals. Additionally, information regarding a physician-diagnosed history of hypertension and elevated cholesterol levels was obtained through self-reported measures.

### 2.6 Statistical analysis

All analyses accounted for the complex survey design of the NHANES by incorporating sampling weights, stratification, and clustering to ensure nationally representative estimates. Data are presented as medians (interquartile ranges [IQRs]) for continuous variables and as unweighted frequencies (weighted percentages) for categorical variables. To examine variations in variable characteristics among groups, we employed the survey-weighted Wilcoxon rank-sum test for continuous variables and the Rao-Scott chi-squared test for weighted percentages of categorical variables, providing a comprehensive description of the entire population. Spearman correlation analyses were conducted to examine the relationships between the HEI-2020 and metabolic indicators.

Binary logistic regression analyses were performed to calculate the odds ratios (ORs) and 95% CIs for the association between HEI-2020 scores and metabolic indicators with NAFLD in participants with T2DM. Three models were constructed: Model 1 was unadjusted; Model 2 adjusted for age and sex; and Model 3 further adjusted for educational level, smoking status, family income-to-poverty ratio, physical activity, body mass index, hypertension, and use of dietary supplements. Notably, race and ethnicity were not adjusted for, as they were included in the calculation of the FLI. To investigate the dose–response relationship between HEI-2020 scores and the prevalence of NAFLD among individuals with T2DM, a restricted cubic spline (RCS) analysis was employed. Furthermore, to evaluate the cumulative influence of the 13 dietary components encompassed within the HEI-2020, the weighted quantile sum (WQS) regression model was implemented (39). In addition, the quantile G-computation (qgcomp) method was applied to assess both the combined and individual contributions of HEI-2020 scores to the risk of NAFLD (40). To enhance the reliability and robustness of the results, the dietary components included in the WQS and qgcomp models were analyzed as separate component scores rather than as a single aggregated score. This methodological approach facilitated a comprehensive examination of the overall health impact of dietary components on NAFLD risk, as well as their specific roles in promoting a balanced dietary pattern. Mediation effects were quantified by calculating the percentage of the indirect effect relative to the total effect, with statistical significance determined using Bootstrap sampling with 1,000 iterations.

Sensitivity analyses were conducted using unweighted, directly weighted, and Taylor-linear adjusted weighted samples for the three study populations. HEI-2020 scores were examined using continuous variables, quartile classifications (Q1, HEI < 34.2; Q2, 43.3 ≤ HEI < 50.8; Q3, 50.8 ≤ HEI < 58.9; Q4, HEI ≥ 58.9), and tertile classifications (T1, HEI < 45.7; T2, 45.7 ≤ HEI < 56.0; T3, HEI ≥ 56.0). The qgcomp model was also used in sensitivity analyses to validate the results from the WQS model. All statistical analyses were performed using R version 4.2.1 (R Foundation) between September 1 and December 31, 2024. A two-tailed *p*-value of <0.05 was considered statistically significant.

## 3 Results

### 3.1 Characteristics of participants

A total of 1,770 confirmed cases of T2DM were included in this study, with a median (IQR) age of 61.00 (51.00–70.75) years. Of the participants, 865 were men (weighted 51.26%), and 646 were non-Hispanic White (weighted 60.06%). The characteristics of T2DM participants, stratified by the presence or absence of NAFLD, are presented in Table 1. Compared to the non-NAFLD group, the NAFLD group had a higher proportion of individuals with a BMI of ≥30, a higher percentage of non-Hispanic White individuals, and a greater prevalence of hypertension. Additionally, the NAFLD group exhibited higher levels of total energy intake, triglycerides, MAP, TyG index, MS, UA, alanine aminotransferase, and aspartate aminotransferase. In contrast, the NAFLD group had lower levels of high-density lipoprotein cholesterol and HEI-2020. There were no significant differences between the two groups regarding education level, family income, smoking status, physical activity, use of dietary supplements, high cholesterol, dyslipidemia, TC, or low-density lipoprotein cholesterol levels.

**Table 1:** Baseline characteristics of participants in NHANES 2007 to 2018.

Characteristic	Overall^1^	Non-NAFLD *n* = 630^1^	NAFLD *n* = 1,140^1^	*p*-value
Age, y				0.325^2^
< 40	136 (9.49%)	39 (8.24%)	97 (10.13%)	
40–60	611 (41.36%)	186 (38.49%)	425 (42.83%)	
≥ 60	1,023 (49.15%)	405 (53.27%)	618 (47.03%)	
Sex				0.260^2^
Male	905 (48.74%)	313 (45.55%)	592 (50.38%)	
Female	865 (51.26%)	317 (54.45%)	548 (49.62%)	
Race and ethnicity				<0.001^2^
Non-Hispanic Black	392 (12.64%)	208 (18.90%)	184 (9.41%)	
Non-Hispanic White	646 (60.61%)	199 (57.10%)	447 (62.41%)	
Mexican American	341 (11.75%)	72 (5.55%)	269 (14.94%)	
Other^4^	391 (15.01%)	151 (18.45%)	240 (13.24%)	
Educational level				0.387^2^
<High school	591 (25.05%)	184 (23.17%)	407 (26.02%)	
High school	417 (26.26%)	157 (24.28%)	260 (27.28%)	
Some college or above	760 (48.69%)	287 (52.55%)	473 (46.70%)	
Family income to poverty ratio				0.429^2^
<1.0	329 (15.60%)	115 (14.06%)	214 (16.38%)	
1.0–3.0	801 (42.97%)	286 (46.46%)	515 (41.19%)	
˃3.0	482 (41.43%)	179 (39.48%)	303 (42.43%)	
Smoking status				0.094^2^
Never	928 (52.14%)	359 (58.14%)	569 (49.04%)	
Former	570 (32.64%)	174 (28.35%)	396 (34.84%)	
Current	272 (15.23%)	97 (13.51%)	175 (16.11%)	
Physical activity				0.543^2^
Light physical activity	941 (49.73%)	337 (48.12%)	604 (50.56%)	
High level of physical activity	829 (50.27%)	293 (51.88%)	536 (49.44%)	
BMI				<0.001^2^
<25	242 (13.20%)	188 (32.33%)	54 (3.34%)	
25–30	521 (27.45%)	247 (39.47%)	274 (21.26%)	
≥30	994 (59.35%)	192 (28.20%)	802 (75.41%)	
Total energy intake, median (IQR), kcal	1,785 (1,337, 2,482)	1,664 (1,357, 2,246)	1,834 (1,336, 2,570)	0.033^3^
Use of dietary supplements				0.736^2^
No	768 (43.13%)	252 (42.27%)	516 (43.57%)	
Yes	1,001 (56.87%)	377 (57.73%)	624 (56.43%)	
Hypertension				0.021^2^
No	667 (39.21%)	257 (45.56%)	410 (35.94%)	
Yes	1,102 (60.79%)	373 (54.44%)	729 (64.06%)	
High cholesterol				0.118^2^
No	686 (40.51%)	272 (44.91%)	414 (38.23%)	
Yes	977 (59.49%)	321 (55.09%)	656 (61.77%)	
Dyslipidemia				0.762^2^
No	140 (6.99%)	45 (6.61%)	95 (7.19%)	
Yes	1,549 (93.01%)	574 (93.39%)	975 (92.81%)	
ALT, median (IQR), U/L	22 (17, 31)	19 (14, 23)	25 (19, 34)	<0.001^3^
AST, median (IQR), U/L	22 (19, 27)	21 (18, 25)	23 (19, 29)	<0.001^3^
TC, median (IQR), mg/dl	182 (152, 211)	179 (150, 208)	182 (152, 213)	0.264^3^
TG, median (IQR), mg/dl	135 (94, 190)	103 (76, 150)	155 (108, 211)	<0.001^3^
LDL, median (IQR), mg/dl	101 (78, 129)	102 (78, 125)	101 (78, 131)	0.676^3^
HDL, median (IQR), mg/dl	45 (39, 55)	50 (43, 64)	44 (38, 51)	<0.001^3^
MAP, median (IQR), mm Hg	90 (83, 99)	89 (81, 97)	91 (84, 99)	0.027^3^
UA, median (IQR), mg/dl	5.60 (4.80, 6.70)	5.00 (4.40, 6.10)	5.90 (5.00, 6.80)	<0.001^3^
TyG, median (IQR)	9.14 (8.73, 9.65)	8.81 (8.43, 9.19)	9.31 (8.95, 9.82)	<0.001^3^
MS, median (IQR)	−0.09 (−1.52, 1.31)	−0.83 (−2.32, 0.36)	0.41 (−1.10, 1.70)	<0.001^3^
HEI-2020, median (IQR)	50 (42, 58)	53 (44, 62)	49 (42, 56)	<0.001^3^

ALT, alanine aminotransferase; AST, aspartate aminotransferase; BMI, body mass index (calculated as weight in kilograms divided by height in meters squared); HDL-C, high-density lipoprotein cholesterol; HEI-2020, Healthy Eating Index 2020; LDL-C, low-density lipoprotein cholesterol; NAFLD, nonalcoholic fatty liver disease; NHANES, National Health and Nutrition Examination Survey; MAP, mean arterial pressure; UA, uric acid; TyG: triglyceride-glucose; MS, metabolic scores; TC, total cholesterol; TG, triglyceride. SI conversion factors: To convert ALT to microkatals per liter, multiply by 0.0167; TC, LDL-C, and HDL-C to millimoles per liter, multiply by 0.0259; TG to millimoles per liter, multiply by 0.0113.

1 All estimates accounted for complex survey designs, and all percentages are weighted.

2 Chi-squared test with Rao & Scott’s second-order correction.

3 Wilcoxon rank-sum test for complex survey samples.

4 Other race and ethnicity includes other Hispanic, other non-Hispanic, and multirace individuals.

### 3.2 Association of HEI-2020 scores with NAFLD in T2DM

**Figure 2:**
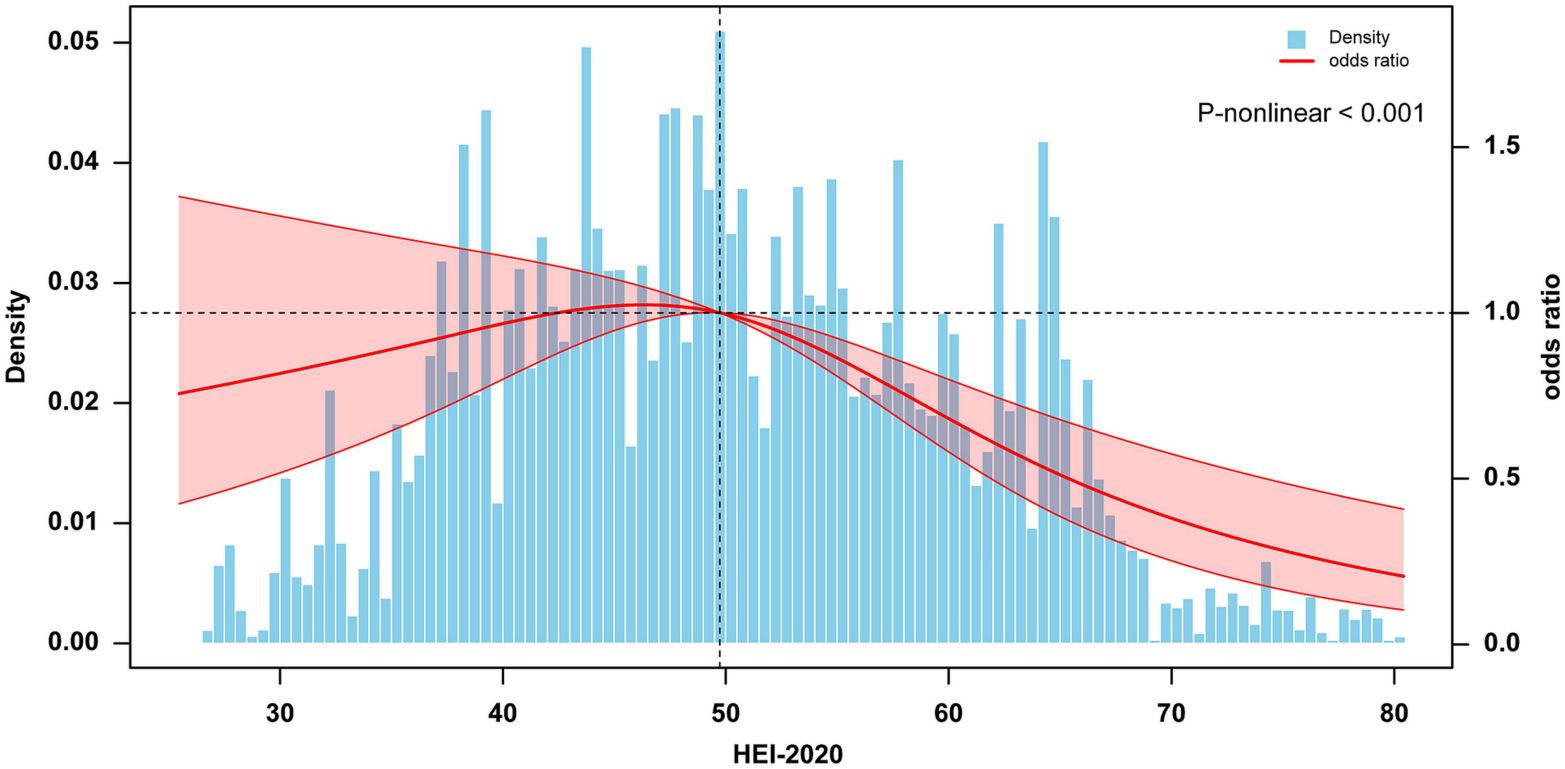
Dose–response association between HEI-2020 (in continues) and NAFLD in T2DM using restricted cubic splines (RCS). The model adjusted for sex, age, educational levels, family income to poverty ratio, smoking status, physical activity, body mass index, hypertension, and use of dietary supplements. NAFLD, nonalcoholic fatty liver disease; T2DM, Type 2 diabetes mellitus; HEI, Healthy Eating Index.

In the comparison of baseline characteristics, we found that the Q2 group (43.3 ≤ HEI < 50.8) and the T2 group (45.7 ≤ HEI < 56.0) had a higher proportion of participants with NAFLD compared to other groups (Supplementary Figure S1). Based on these findings, we selected the Q2 and T2 groups as reference groups and constructed a multivariable stepwise logistic regression model to explore the association between HEI-2020 and NAFLD. Table 2 presents the association between higher HEI-2020 scores and lower NAFLD risk in T2DM patients across three binary logistic regression models. Regardless of whether the study sample was weighted, HEI-2020 scores as a continuous variable showed a significant health impact on NAFLD risk in T2DM patients (OR 0.978, 95% CI: 0.959–0.998). Additionally, when HEI-2020 scores were categorized into tertiles and quartiles, this association remained significant (tertiles: OR 0.451, 95% CI: 0.270–0.752; quartiles: OR 0.469, 95% CI: 0.270–0.814). The RCS results showed a negative dose-relative relationship between HEI and the risk of NAFLD in Figure 2.

**Table 2:** Relationship between HEI-2020 with NAFLD in T2DM.

Variable	OR (95%CI)		
	Unweighted	Crude weighted	Adjusted Weighted
Model 1^1^			
HEI continuous	0.971 (0.962, 0.979)^*^	0.966 (0.951, 0.981)^*^	0.966 (0.950, 0.983)^*^
HEI Category (ref T2)			
T1	1.098 (0.860, 1.403)	0.779 (0.526, 1.152)	0.779 (0.531, 1.142)
T3	0.662 (0.522, 0.839)^*^	0.415 (0.285, 0.606)^*^	0.415 (0.264, 0.652)^*^
HEI Category (ref Q2)			
Q1	1.153 (0.866, 1.536)	0.935 (0.581, 1.506)	0.935 (0.576, 1.519)
Q3	0.850 (0.643, 1.124)	0.739 (0.471, 1.160)	0.739 (0.490, 1.117)
Q4	0.549 (0.417, 0.721)^*^	0.408 (0.257, 0.647)^*^	0.408 (0.250, 0.665)^*^
Model 2^2^			
HEI continuous	0.973 (0.964, 0.981)^*^	0.967 (0.952, 0.983)^*^	0.967 (0.950, 0.984)^*^
HEI Category (ref T2)			
T1	1.071 (0.838, 1.370)	0.770 (0.521, 1.136)	1.300 (0.881, 1.916)
T3	0.686 (0.540, 0.871)^*^	0.427 (0.291, 0.627)^*^	0.555 (0.358, 0.859)^*^
HEI Category (ref Q2)			
Q1	1.133 (0.850, 1.513)	0.921 (0.575, 1.473)	0.921 (0.567, 1.495)
Q3	0.865 (0.653, 1.144)	0.746 (0.474, 1.172)	0.746 (0.490, 1.134)
Q4	0.574 (0.435, 0.756)^*^	0.420 (0.264, 0.670)^*^	0.420 (0.255, 0.692)^*^
Model 3^3^			
HEI continuous	0.984 (0.973, 0.995)^*^	0.978 (0.960, 0.997)^*^	0.978 (0.959, 0.998)^*^
HEI Category (ref T2)			
T1	0.859 (0.643, 1.146)	0.650 (0.419, 1.008)	0.650 (0.409, 1.030)
T3	0.718 (0.541, 0.952)^*^	0.451 (0.292, 0.695)^*^	0.451 (0.270, 0.752)^*^
HEI Category (ref Q2)			
Q1	0.957 (0.687, 1.334)	0.695 (0.418, 1.156)	0.695 (0.397, 1.218)
Q3	0.967 (0.697, 1.343)	0.783 (0.482, 1.271)	0.783 (0.507, 1.208)
Q4	0.684 (0.494, 0.947)^*^	0.469 (0.284, 0.774)^*^	0.469 (0.270, 0.814)^*^

NAFLD, nonalcoholic fatty liver disease; T2DM, Type 2 diabetes mellitus; OR, odds ratio; CI, confidence interval; HEI, Healthy Eating Index; T, Tertile; Q, Quartile.

* *p < 0.05, Crude weighed = add weight to unweighted. Adjusted weighted = Taylor line correction for Crude weighted.*

1 Crude model.

2 Adjusted for age and sex.

3 Further adjusted for educational levels, family income to poverty ratio, smoking status, physical activity, body mass index, hypertension, and use of dietary supplements.

### 3.3 Mixed effects of 13 dietary components on NAFLD in T2DM

**Figure 3:**
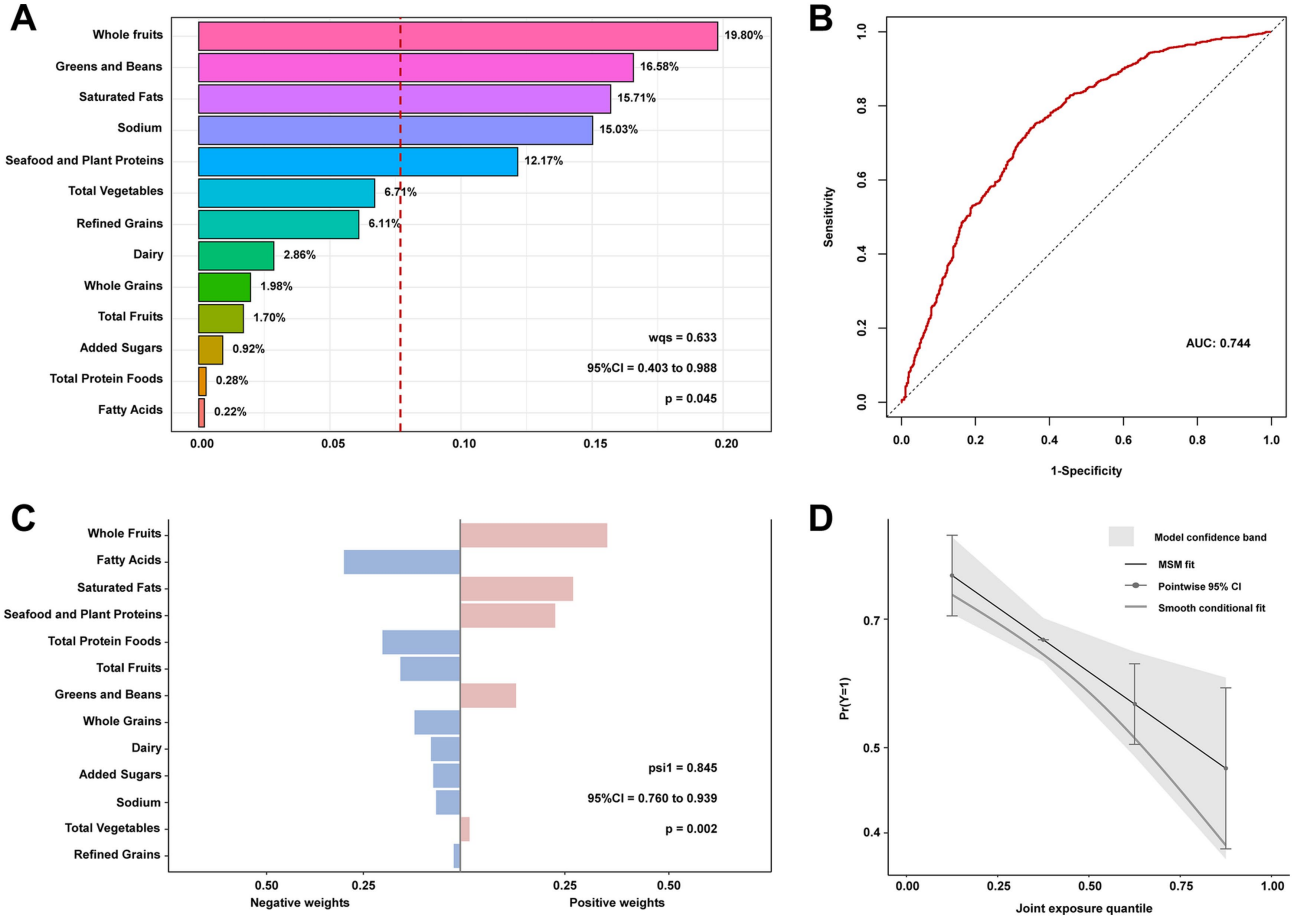
Contribution weights of dietary components in the positive direction WQS model of NAFLD in T2DM **(A)** and the AUCs of the WQS models **(B)**. Contribution weights of dietary components in the qgcomp model of NAFLD in T2DM **(C)** and the trends visualization of qgcomp model **(D)**. All models adjusted for sex, age, educational levels, family income to poverty ratio, smoking status, physical activity, body mass index, hypertension, and use of dietary supplements. NAFLD, nonalcoholic fatty liver disease; T2DM, Type 2 diabetes mellitus; WQS, weighted quantile sum; AUCs, area under curves.

The results from the WQS model indicate that HEI-2020 scores exhibit a significant protective effect on NAFLD risk in individuals with T2DM when treated as a composite variable (WQS index OR 0.633, 95% CI: 0.403–0.988, *p* = 0.045). The 13 dietary components of HEI-2020 collectively demonstrate a beneficial combined effect on NAFLD risk (Figure 3). Using 1/13 as the reference standard for WQS analysis, the WQS positive weight analysis identified Whole Fruits, Greens and Beans, and Saturated Fat as the top three key contributors mediating this protective effect, with weights of 19.80, 16.58, and 15.71%, respectively. These components were associated with a reduced risk of NAFLD, highlighting their potential role in mitigating disease progression. Furthermore, the WQS negative weight analysis corroborated the findings from the positive weight analysis (Supplementary Figure S3). The weights of the individual HEI-2020 components, as determined by the qgcomp method, were consistent with those derived from the WQS regression results. The qgcomp model showed a significant negative association between HEI-2020 and NAFLD risk (OR 0.845, 95% CI: 0.760–0.939, *p* = 0.002).

### 3.4 Correlations between HEI-2020 and metabolism indicators and the association of metabolism indicators with NAFLD in T2DM

Correlation analysis of the HEI-2020 with metabolic indicators across all study participants demonstrated a significant inverse association between HEI-2020 scores and these metabolic markers. The most pronounced negative correlations were identified between HEI-2020 and the TyG index (r = −0.12) and MS (r = −0.13). Comparable patterns were evident in subgroup analyses stratified by NAFLD and non-NAFLD status, as illustrated in Supplementary Figure S3. Among all the metabolic dysfunction indicators, UA (OR 1.29, 95% CI: 1.13–1.47, *p* < 0.001), TyG index (OR 3.98, 95% CI: 2.91–5.45, *p* < 0.001), and MS (OR 1.35, 95% CI: 1.23–1.48, *p* < 0.001) exhibited significant associations with NAFLD in individuals with T2DM (Supplementary Figure S4).

### 3.5 Mediation effects of metabolic indicators on the associations of HEI-2020 with NAFLD in T2DM

**Figure 4:**
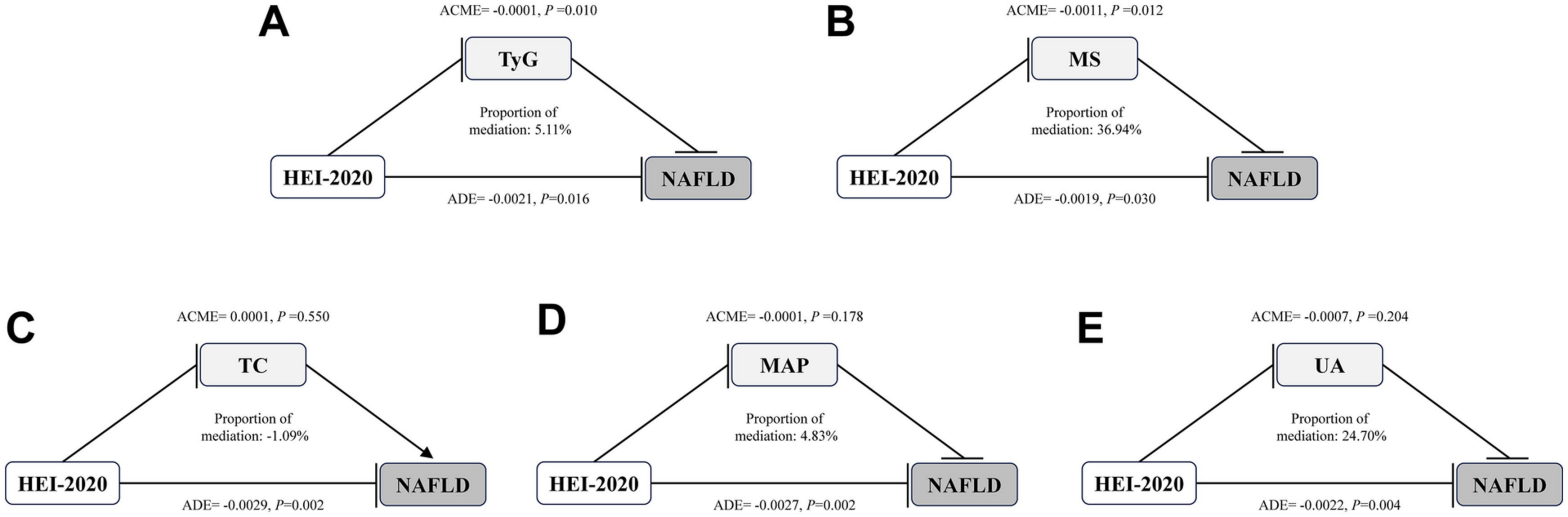
Mediation effects of metabolic indicators on the associations of HEI-2020 with NAFLD in T2DM. Arrows and rounded heads indicate promotion and inhibition, respectively. NAFLD, nonalcoholic fatty liver disease; T2DM, Type 2 diabetes mellitus; HEI, Healthy Eating Index; TC, total cholesterol; MAP, mean arterial pressure; UA, uric acid; TyG: triglyceride-glucose; MS, metabolic scores; ACME, average causal mediation effects; ADE, average direct effects.

A mediation effect analysis was conducted to explore the relationships among dietary quality (as measured by HEI-2020), metabolic parameters (including UA, MAP, TC, TyG and MS), and NAFLD. The results indicated that TyG and MS mediated 5.11 and 36.94% of the association between HEI-2020 and NAFLD, respectively (Figure 4).

## 4 Discussion

This research presents a novel investigation into the association between the HEI-2020 and the risk of NAFLD in individuals diagnosed with T2DM, highlighting the potential protective effect of improved dietary quality. Furthermore, metabolic dysfunction indicators, including MS and the TyG index, was identified as a partial mediator in this relationship. Higher HEI-2020 scores were significantly correlated with a decreased risk of NAFLD among T2DM patients, even after accounting for multiple confounding factors. The most pronounced protective effects were observed in the highest quartiles of dietary quality. Among the 13 dietary components assessed within the HEI-2020 framework, whole fruits emerged as the most significant contributor to reducing NAFLD risk.

Notably, the current study extends these findings by focusing specifically on patients with T2DM, a population at higher risk for both NAFLD and other comorbidities, such as hypertension and dyslipidemia. A study by Swaminathan et al. demonstrated that adherence to dietary guidelines plays a protective role in metabolic diseases, including T2DM (41). Our findings align with previous research highlighting the protective effects of dietary patterns on NAFLD risk. For instance, several studies have consistently indicated that high-quality diets, particularly those with greater adherence to dietary guidelines, are associated with a reduced incidence of NAFLD in both the general population and individuals with metabolic disorders, including T2DM (13, 42, 43). By specifically focusing on the T2DM population, our study further underscores the importance of diet in this group, which is characterized by a higher prevalence of comorbidities.

The association between the HEI-2020 and NAFLD was further substantiated through dose–response analysis, which demonstrated a significant inverse relationship between diet quality and the risk of developing NAFLD. A comprehensive prospective cohort study conducted using data from the UK Biobank identified that dietary scoring systems, including the Recommended Food Score (RFS), the 14-Item Mediterranean Diet Adherence Screener (MEDAS-14), and the Healthy Diet Indicator (HDI), were among the most robust predictors of severe NAFLD. Across various dietary assessment methodologies, adherence to a healthy diet consistently emerged as a protective factor against the development of NAFLD (44). Additionally, both the Dietary Approaches to Stop Hypertension (DASH) diet and the Mediterranean diet were linked to a decreased risk of metabolic-associated fatty liver disease (MAFLD) in overweight and obese populations. A randomized controlled trial highlighted that the DASH diet was effective in reducing body weight and mitigating metabolic risk factors, such as insulin resistance, triglycerides, serum liver enzymes, and inflammatory biomarkers, in overweight or obese individuals with MAFLD (45). Similarly, when compared to a conventional diet, the Mediterranean diet was shown to enhance insulin sensitivity, improve blood lipid profiles, and reduce liver fat accumulation in overweight patients with MAFLD (46). Although several studies have explored the relationship between diet and NAFLD, few have specifically examined the role of HEI-2020 in T2DM populations. Our findings further support existing literature indicating that better diet quality, as reflected in higher HEI scores, is associated with a reduced risk of chronic diseases, including NAFLD (9, 47, 48).

This study further explored the impact of dietary components on NAFLD. WQS regression analysis identified whole fruits, greens and beans, and saturated fat as the top three dietary components contributing to the protective effect of the HEI-2020 against NAFLD. These findings align with previous research emphasizing the benefits of plant-based diets in managing metabolic disorders (47, 49). For instance, a study by Xia et al. demonstrated that adherence to a vegetable-based diet could reduce the risk of NAFLD in patients with T2DM (48). This suggests that improving dietary patterns, particularly increasing the intake of whole fruits, and greens, and reducing saturated fats, could play a key role in preventing or managing NAFLD in T2DM patients.

Additionally, the mediating roles of metabolic indicators, such as the TyG index and metabolic score, were identified, highlighting the complex interplay between diet, metabolic health, and liver function. This insight builds on earlier work suggesting that diet may be associated with liver health through intermediary metabolic pathways, such as insulin resistance and dyslipidemia (8, 50). Notably, recent Mendelian randomization studies have linked micronutrient and macronutrient intake to the risk of allergic diseases, underscoring the complexity of dietary effects on inflammation and immunity (51). Furthermore, several studies have investigated the relationship between nutritional biomarkers and metabolic indicators (52, 53). The metabolic score, calculated using multiple metabolic indicators, serves as a marker of metabolic dysfunction. Higher MS values indicate more severe metabolic dysfunction. Consistent with recent studies, our research also found significant associations between metabolic indicators, particularly the MS and TyG index, and the risk of NAFLD in patients with T2DM. These findings align with earlier studies that have proposed the TyG index as a reliable marker of insulin resistance and its association with liver fat accumulation (23, 24). However, the current study provides new insights into how diet quality, through its impact on these metabolic indicators, may mitigate NAFLD progression, further underscoring the importance of dietary interventions in managing metabolic diseases.

One notable strength of this research lies in its utilization of the NHANES dataset, a comprehensive and nationally representative sample, which significantly enhances the generalizability of the findings. Moreover, the application of multiple regression models, complemented by sensitivity analyses and the qgcomp method, facilitated a thorough and robust examination of the relationships between the HEI-2020 and NAFLD among individuals with T2DM. Nevertheless, this study is not without its limitations. The cross-sectional nature of the NHANES survey inherently restricts the ability to establish causal relationships. Additionally, the reliance on self-reported dietary data, despite the use of the validated Automated Multiple-Pass Method for data collection, may introduce potential biases. Furthermore, due to the availability of imaging results only for certain years within the NHANES 2007–2018 dataset, this study used the FLI as a diagnostic tool for NAFLD. While FLI has demonstrated good accuracy as a non-invasive measure, it is not based on imaging findings and may not identify all affected patients. The study also accounted for several confounding variables, but did not consider other influential factors such as gut microbiota composition or genetic predispositions to NAFLD, which could potentially impact the results. These limitations highlight the need for further research to address these gaps and validate the findings.

## 5 Conclusion

In conclusion, this study tested the hypothesis that higher adherence to the HEI-2020 is associated with a lower likelihood of NAFLD in T2DM patients, with metabolic indicators mediating this relationship. The findings underscore the importance of improving dietary quality as a key strategy in managing liver health and metabolic dysfunction in this high-risk population. Public health initiatives and dietary interventions focusing on promoting better adherence to the HEI-2020 could significantly reduce the burden of NAFLD in individuals with T2DM. These efforts should be prioritized to enhance metabolic health and prevent liver-related complications, particularly in vulnerable populations.

## Data Availability

Publicly available datasets were analyzed in this study. This data can be found here: the data used in this study are publicly available from the National Health and Nutrition Examination Survey (NHANES) for the years 2008–2018. The dataset can be accessed at https://wwwn.cdc.gov/nchs/nhanes/default.aspx. All analyses were performed on these publicly accessible data in compliance with ethical standards.
