# Supplementary material for: Associations of healthy eating index 2020 and its components with non-alcoholic fatty liver disease in type 2 diabetes patients and the mediating roles of metabolic indicators: NHANES 2007–2018
Source: Front Nutr. 2025 Apr 9;12:1564197. doi: 10.3389/fnut.2025.1564197 (PMC12014457; doi:10.3389/fnut.2025.1564197)

Associations of Healthy Eating Index 2020 and its components with non-alcoholic fatty liver disease in type 2 diabetes patients and the mediating roles of metabolic indicators: NHANES 2007–2018

Jingxiong Chen[1, 2†], Haizhou Diao[3†], Yuling Zhang[4†], Ben Hu[5], Kai Qian[2], Kaiguang Zhang[2], Tengyue Zhang[3*], Jizhong Song[1,2*]

[1]Department of Postgraduates, Bengbu Medical University, Bengbu, 233030, Anhui, China.

[2]Department of Gastroenterology, the First Affiliated Hospital of USTC, Division of Life Sciences and Medicine, University of Science and Technology of China, Hefei, Anhui, 230031, China.

[3]Department of Oncology, The First Affiliated Hospital of USTC, Division of Life Sciences and Medicine, University of Science and Technology of China, Hefei, Anhui, 230031, China.

[4]Department of Gastrointestinal Surgery, The First Affiliated Hospital of USTC, Division of Life Sciences and Medicine, University of Science and Technology of China, Hefei, Anhui, 230031, China.

[5]Department of Cardiology, The Second People's Hospital of Hefei, Hefei Hospital Affiliated to Anhui Medical University, Hefei, 230011, Anhui, China.

Jingxiong Chen[1,2 †],Haizhou Diao[3, †], Yuling Zhang[4, †] These authors contributed equally to this work and share first authorship.

Table of Contents

Figure S1. Comparison of the proportion of NAFLD in T2DM between groups (A)based on HEI-2020 quartiles and (B)based on HEI-2020 tertiles. Use weighted sample size to calculate.

Figure S2. Contribution weights of dietary components in the negative direction WQS model of NAFLD in T2DM.

Figure S3. Spearman's rank correlation coefficients between HEI-2020 and metabolic indices.

Figure S4. Associations of metabolic indicators with NAFLD in T2DM.

Figure S1. Comparison of the proportion of NAFLD in T2DM between groups (A)based on HEI-2020 quartiles and (B)based on HEI-2020 tertiles. Use weighted sample size to calculate.

Abbreviations: NAFLD, nonalcoholic fatty liver disease; T2DM, Type 2 diabetes mellitus; HEI, Healthy Eating Index

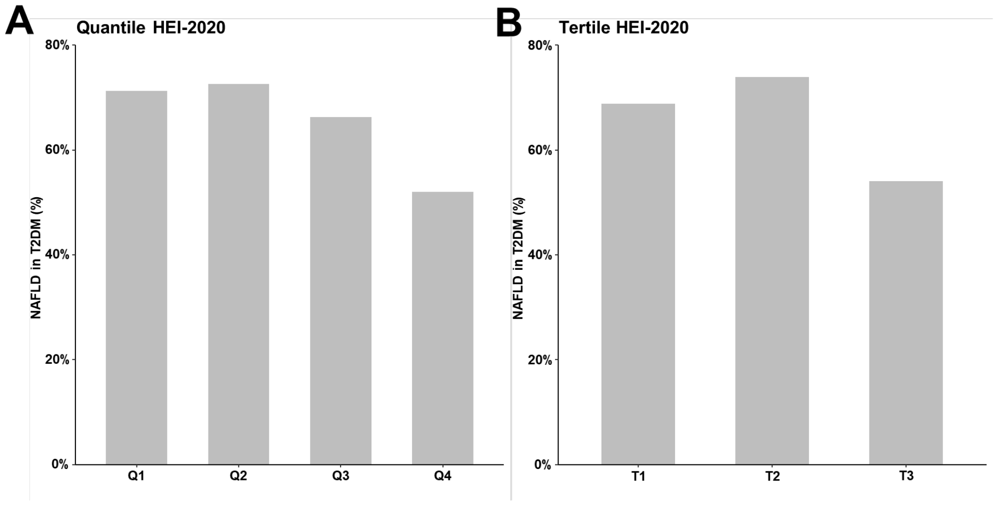

Figure S2. Contribution weights of dietary components in the negative direction WQS model of NAFLD in T2DM

Abbreviations: NAFLD, nonalcoholic fatty liver disease; T2DM, Type 2 diabetes mellitus; WQS, weighted quantile sum

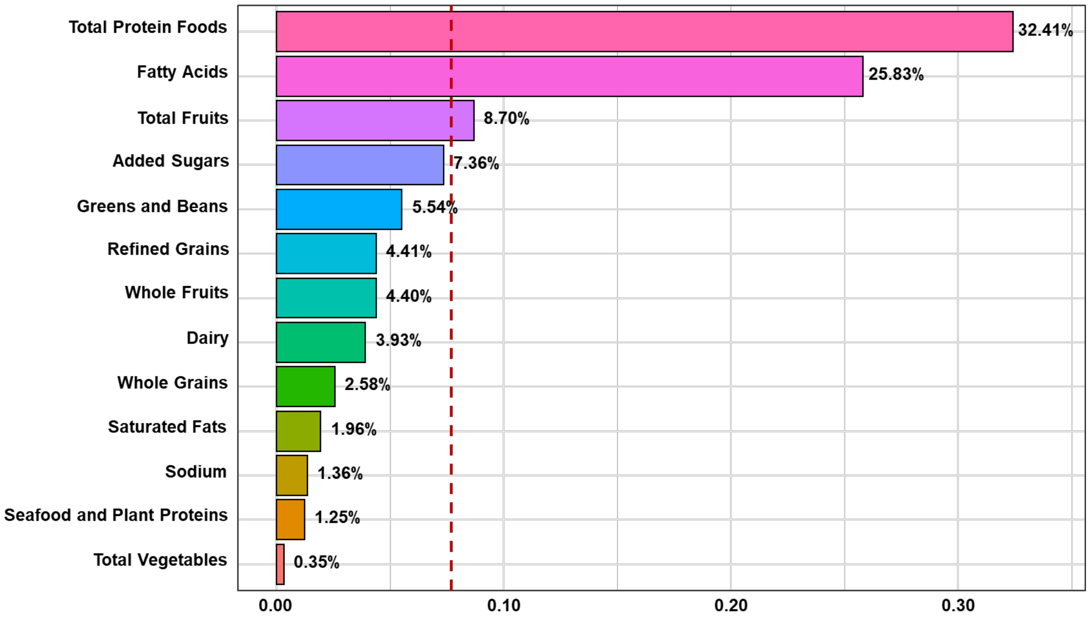

Figure S3. Spearman's rank correlation coefficients between HEI-2020 and metabolic indices.

Abbreviations: NAFLD, nonalcoholic fatty liver disease; HEI, Healthy Eating Index; MAP, mean arterial pressure; UA, uric acid; TyG: triglyceride-glucose; MS, metabolic scores

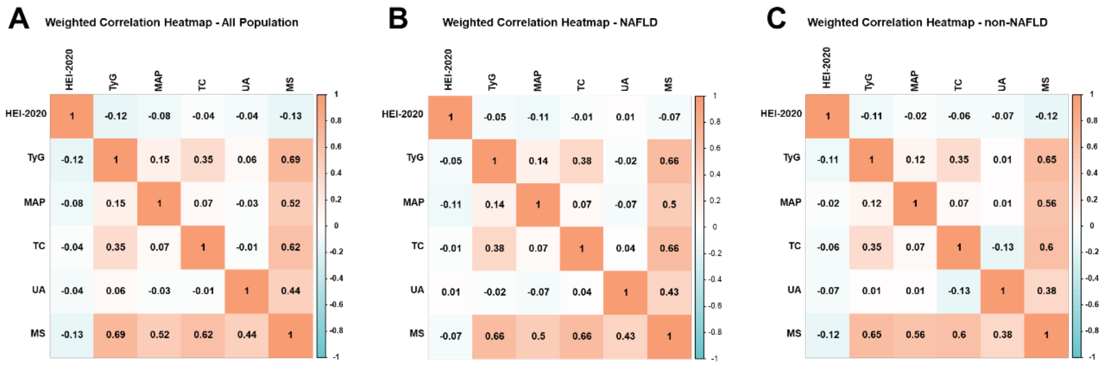

Figure S5. Associations of metabolic indicators with NAFLD in T2DM.

Abbreviations: NAFLD, nonalcoholic fatty liver disease; T2DM, Type 2 diabetes mellitus; MAP, mean arterial pressure; UA, uric acid; TyG: triglyceride-glucose; MS, metabolic scores; OR, odds ratio; CI, confidence interval

a Crude model.

b Adjusted for age and sex.

c Further adjusted for educational levels, family income to poverty ratio, smoking status, physical activity, body mass index, hypertension, and use of dietary supplements.

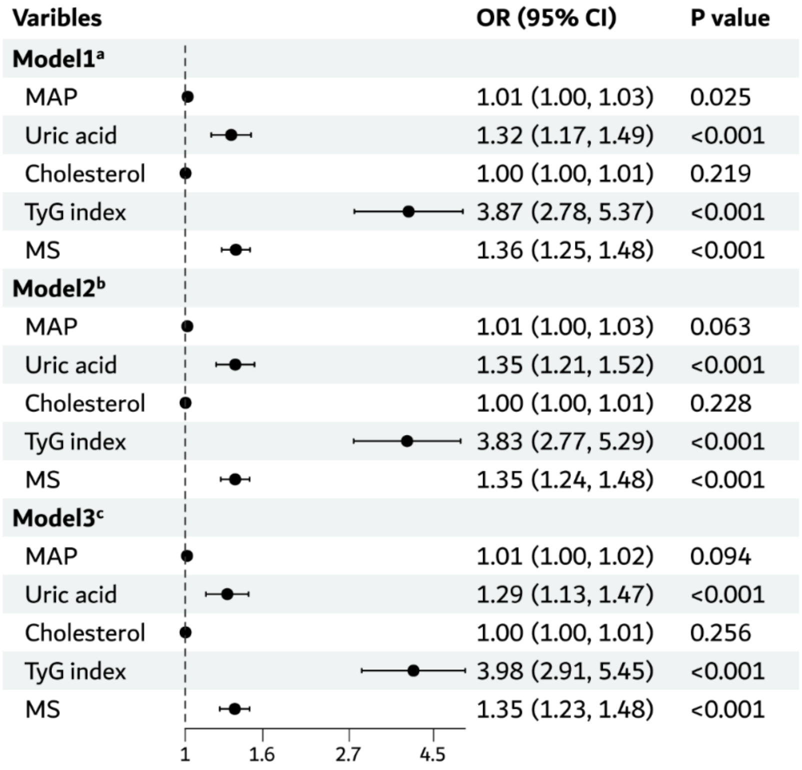

Supplement: Supplementary file 1 [file Image_1.pdf]
